# Supplementary material for: EMT, Stemness, and Drug Resistance in Biological Context: A 3D Tumor Tissue/In Silico Platform for Analysis of Combinatorial Treatment in NSCLC with Aggressive KRAS-Biomarker Signatures
Source: Cancers (Basel). 2022 Apr 27;14(9):2176. doi: 10.3390/cancers14092176 (PMC9099837; doi:10.3390/cancers14092176)
Supplement: Supplementary file 1 [file cancers-14-02176-s001.zip › cancers-1583075-supplementary.pdf]

cancers

MDPI

Supplementary Material: EMT, Stemness, and Drug Resistance in Biological Context: A 3D Tumor Tissue/In Silico Platform for Analysis of Combinatorial Treatment in NSCLC with Aggressive KRAS-Biomarker Signatures

Matthias Peindl, Claudia Göttlich, Samantha Crouch, Niklas Hoff, Tamara Lüttgens, Franziska Schmitt, Jesús Guillermo Nieves Pereira, Celina May, Anna Schliermann, Corinna Kronenthaler, Danjouma Cheufou, Simone Reu-Hofer, Andreas Rosenwald, Elena Weigl, Thorsten Walles, Julia Schüler, Thomas Dandekar*, Sarah Nietzer and Gudrun Dandekar*

Supplemental Material

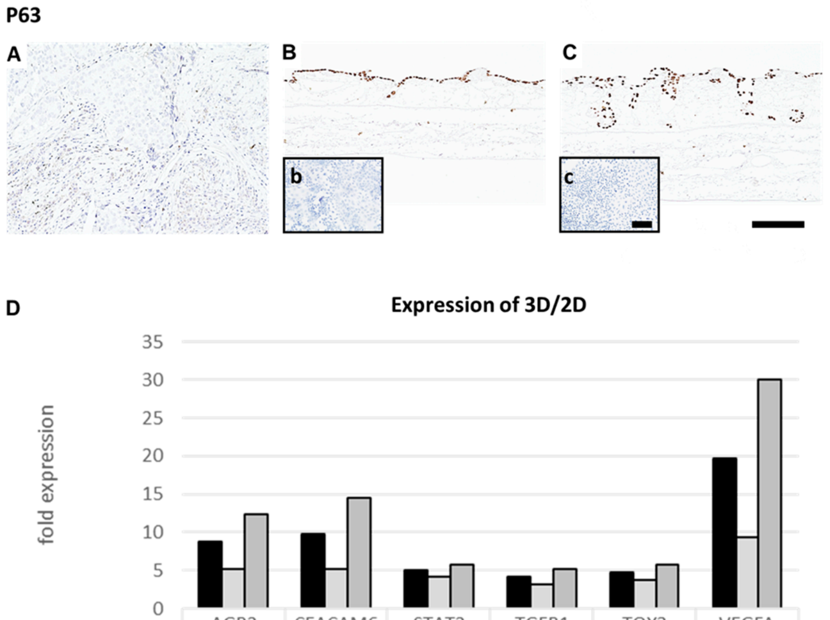

	AGR2	CEACAM6	STAT2	TGFB1	TOX3	VEGFA
Mean	9	10	5	4	5	20
experiment 1	5	5	4	3	4	9
experiment 2	12	14	6	5	6	30

Figure S1. Differences between 2D and 3D culture. The clinical marker P63 for squamous carcinomas is also slightly expressed in clinical specimens of an adenocarcinoma (A) and is strongly upregulated under 3D culture conditions in HCC827 (B) and A549 cells (C). D: fold increase of expression in 3D compared to 2D cell culture of HCC827 cells in two different experiments ($n = 2$). Scale bar in C = 100 µm for (**A-C**); scale bar in c = 100 µm for (**b and c**).

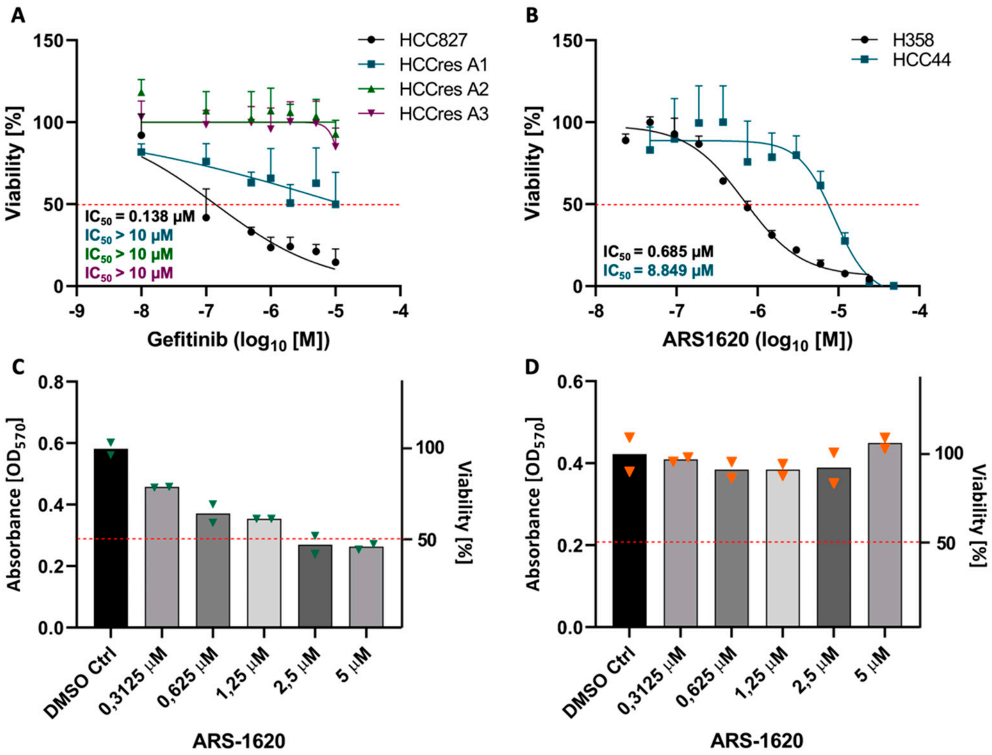

Figure S2. Differences between 2D and 3D culture: Drug testing of EGFR- or $KRAS^{G12C}$-mutated cells. 2D CellTiter Glo viability assay of (A) HCC827 cells and resistant subpopulations A1, A2 and A3 treated with increasing concentrations of gefitinib ($n \geq 3$) or (B) H358 and HCC44 cells treated with increasing concentrations of ARS-1620; $n = 1$ with 3 technical replicates. MTT assays of (C) H358 or (D) HCC44 3D tumor models, treated with different concentrations of ARS-1620 for 72 hours ($n = 2$). Red line marks 50% viability. Triangles (▼) represent values from single biological replicates.

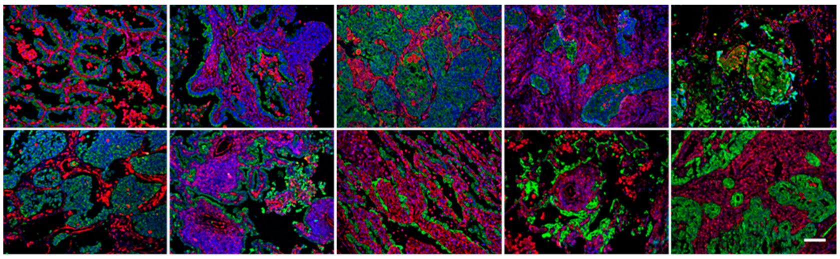

Figure S3. Immunofluorescence staining of lung adenocarcinomas. Pan-cytokeratin (green) and vimentin (red) staining with DAPI (blue) counterstaining of 10 clinical lung adenocarcincoma specimens; scale bar = 100 µm.

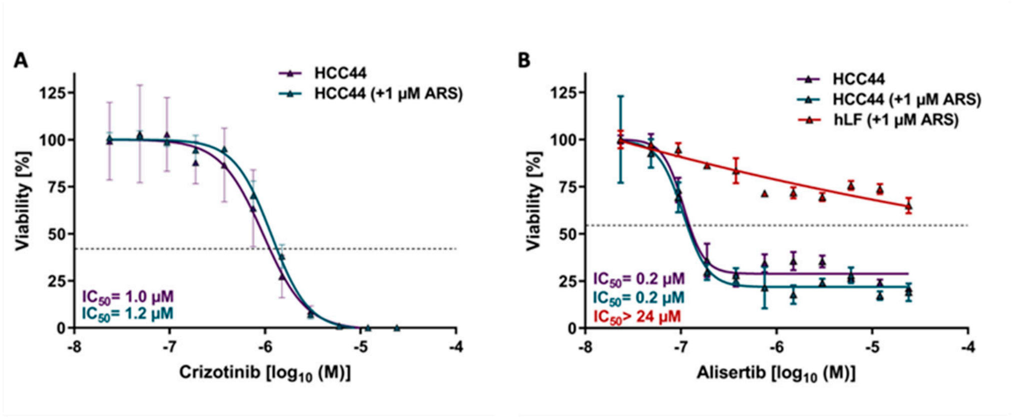

Figure S4. Combination of ARS-1620 with crizotinib or alisertib in 2D**.** (A) CellTiter-Glo® viability assay of HCC44 cells treated with increasing concentrations of crizotinib. **(B)** CellTiter-Glo® viability assay of HCC44 cells and primary lung fibroblasts (hLF) treated with alisertib. Cells were treated with a monotherapy of the indicated drug or in a constant combination with 1 µM ARS-1620; *n* = 1 with three technical replicates.

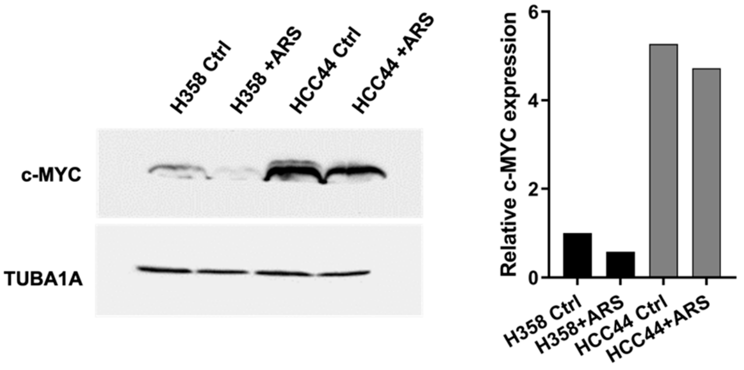

Figure S5. Expression of c-MYC in H358 and HCC44 cells in 3D. Western blot with quantification showing the expression of c-MYC in H358 and HCC44 cells cultured on the SISmuc for 14 days. The models were treated with 1 µM ARS-1620 for the last 72 hours in culture. Alpha-tubulin (TUBA1A) was used as a loading control; *n* = 1.

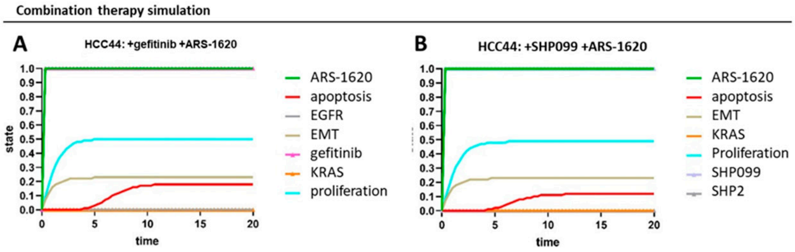

Figure S6. In silico combination therapy simulations of HCC44 cell line. (A) Simulation of HCC 44 cells treated with gefitinib and ARS-1620. (B) HCC44 treated with ARS-1620 combined with SHP099. Color code for different read-out parameters is given in the legend. Both simulations show no drug response.

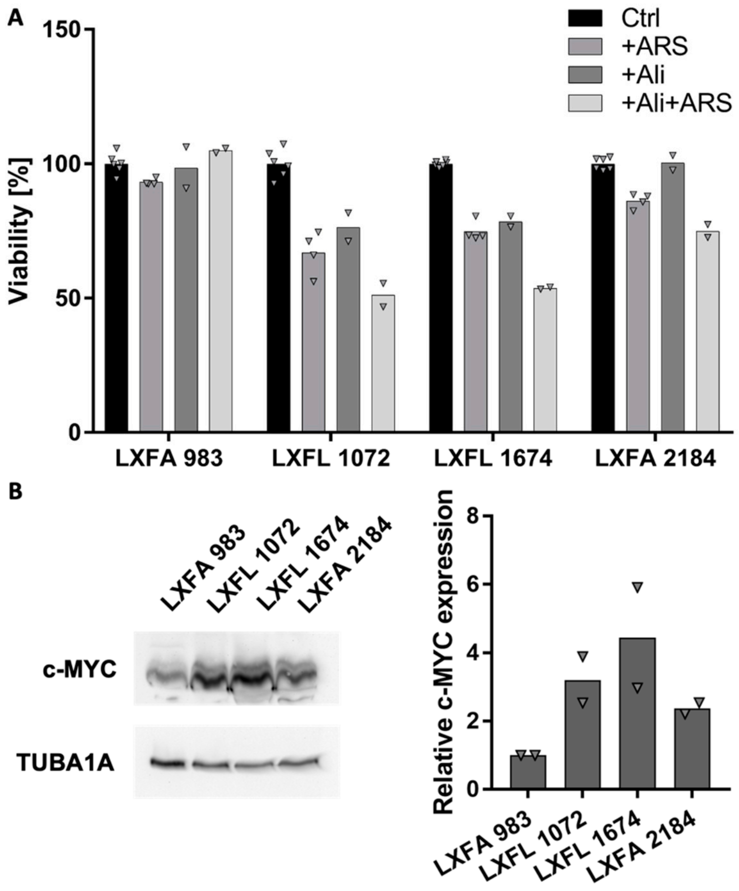

Figure S7. Combination of alisertib and ARS-1620 in PDX-cell lines with differential c–MYC expression. (A) Viability determined by MTT assay of the PDX derived cell lines LXFA 983, LXFL 1072, LXFL 1674 and LXFA 2184 on 3D SISmuc tumor models after treatment with either 1 µM ARS-1620, 5 µM alisertib or a combination of both inhibitors for 72 hours; $2 \leq n \leq 4$. (B) Western blot with quantification showing the expression of c-MYC in the PDX derived cell lines. Cells were cultured on SISmuc tumor models for 14 days. Alpha-tubulin (TUBA1A) was used as loading control; $n = 2$. Triangles (▼) represent values from single biological replicates.

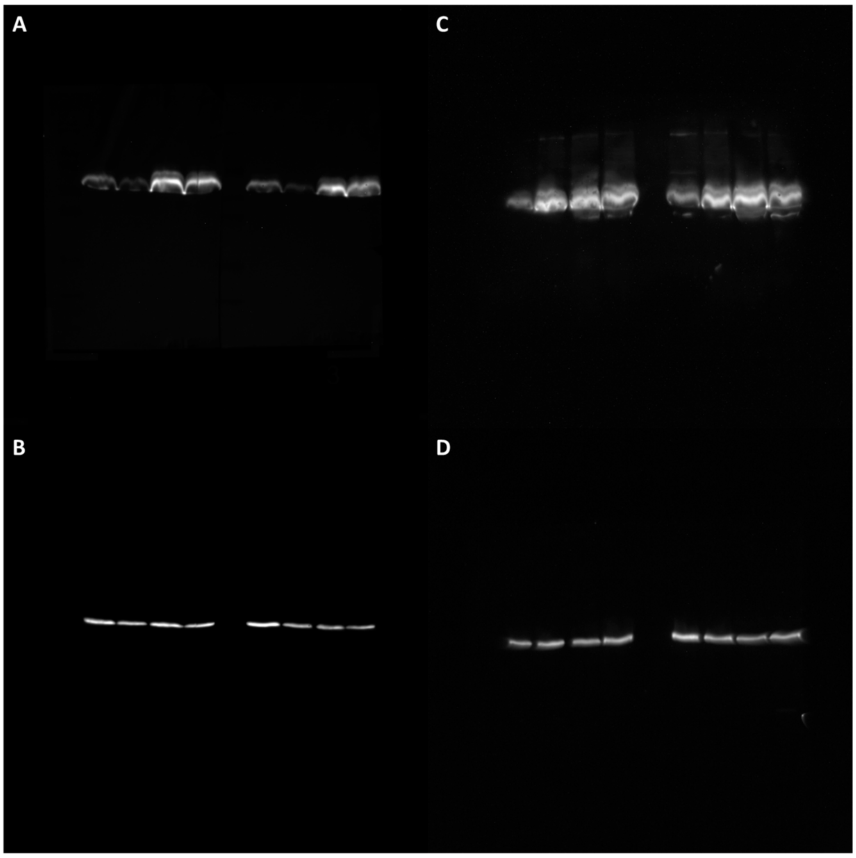

Figure S8. Original, uncropped images of c-MYC Western Blots with loading controls. (A) c-MYC Western Blot of H358 and HCC44 cells in 3D with or without ARS-1620 treatment and (B) alpha-Tubulin as loading control. Sample order according to Figure S5. (C) c-MYC Western Blot of LXFA 983, LXFL 1072, LXFL 1674 and LXFA2184 cells in 3D and (D) alpha-Tubulin as loading control. Sample order according to Figure S7.

Table S1. Simulation parameters at start (ground state) of the simulation of the KRAS^{G12C}-biomarker-model, untreated (gray) and with ARS-1620 treatment (white).

A: ground state[1]	H358 untreated	H358 treated with ARS-1620	HCC44 untreated	HCC44 treated with ARS-1620
**ARS-1620**	0	1	0	1
**E-cadherin**	0.300	0.290	0.100	0.100
**MUC-1**	0.300	0.300	0.150	0.145
**vimentin**	0.250	0.240	0.350	0.340
**CD44**	0.050	0.050	0.250	0.250
(KRAS)	0.400	0.400	0.400	0.400
HGFR (MET)	0.200	0.180	0.200	0.200
AKT/PKB	0.250	0.230	0.250	0.240
AMPK	0.660	0.660	0.660	0.660
**c-Myc**	0.200	0.100	0.350	0.340
ERK	0.290	0.260	0.290	0.285
MEK	0.300	0.280	0.300	0.295
SNAIL	0.300	0.290	0.200	0.190
ERK1	0.280	0.250	0.280	0.275
ERK2	0.150	0.130	0.150	0.145
PI3K	0.300	0.280	0.300	0.280
RAF	0.270	0.250	0.270	0.265
KEAP1	0.680	0.680	0.400	0.400

AURKA	0.100	0.090	0.100	0.090
ATR	0.780	0.790	0.780	0.790
GSK-3ß	0.430	0.450	0.530	0.520
**B: outcome[2]**				
**proliferation**	0.290	0.240	0.500	0.480
**apoptosis**	0.120	0.940	0.030	0.120
**EMT**	0.080	0.070	0.240	0.220

[1] (A) Ground state activities for the different nodes in our model that were not zero; these parameters serve to predict the model responses correctly according to the experimental data though only a core network of central pathways is modelled (in the cell there would be 5.000 proteins present, we calculate only for very few central nodes). Color code: gray (pre-stimulation for treatment simulation), red font: show largest differences between both cell lines. Experimentally measured parameters are shown in bold. [2] (B) Outcome (effector output): Experimentally measured parameters are shown in bold. All other nodes of the network were at zero activity at simulation start.

Table S2. Simulation parameters at start of the simulation (ground state) for indicated individual targeted combination therapy of the HCC44 KRAS^{G12C}-biomarker-model in combination with ARS-1620).

A ground state[1.]	crizotinib	SHP099	gefitinib	alisertib
**ARS-1620**	1	1	1	1
**E-cadherin**	0.100	0.100	0.100	0.120
**MUC-1**	0.145	0.145	0.145	0.145
**vimentin**	0.340	0.340	0.340	0.280
**CD44**	0.250	0.250	0.250	0.250
(KRAS)	0.400	0.400	0.400	0.400
HGFR (MET)	0.200	0.200	0.200	0.200
AKT/PKB	0.230	0.230	0.240	0.240
AMPK	0.660	0.660	0.660	0.650
c-Myc	0.340	0.340	0.330	0.340
ERK	0.285	0.285	0.260	0.285
MEK	0.295	0.295	0.270	0.295
SNAIL	0.190	0.190	0.190	0.190
ERK1	0.275	0.275	0.25	0.275
ERK2	0.145	0.145	0.13	0.145
PI3K	0.270	0.270	0.270	0.280
RAF	0.265	0.265	0.250	0.265
KEAP1	0.400	0.400	0.400	0.400
AURKA	0.090	0.090	0.090	---
ATR	0.790	0.790	0.790	0.790
GSK-3β	0.520	0.520	0.550	0.540
**B outcome[2]**				
**proliferation**	0.480	0.480	0.500	0.480
**apoptosis**	0.120	0.120	0.180	0.460
**EMT**	0.220	0.220	0.180	0.160

[1] (A) The software allows to calculate now the effects of any combination therapy of choice including triple or multiple therapies, from the ground state of individual therapies. Similarly, the activity for every node in the network and its changes over time are modelled but only selected ones are shown and plotted in the figures. Ground state activities for the different nodes in our model; these parameters serve to predict the model responses correctly according to the experimental data though only the core network is modelled. Blue fields: additional modification of the ground state compared to Table S1 to simulate the action of combination treatment correctly. **General Method:** The simulation results without the prediction of the alisertib / ARS-1620 combination were generated based on experimental results. Here, the ground state of the HCC44 before and after ARS-1620 treatment (Table S1) and also the three other combinatory treatment simulations SHP099, crizotinib, and gefitinib served for adjustment and control of the network (Table S2). H358 is sensitive to the ARS-1620 treatment and should illustrate the response of a sensitive cell

line and its ground state settings compared to the resistant HCC44 cell line. For the combinatorial simulation, the condition after treatment of HCC44 with ARS-1620 was used. Based on this, additional values, which were assumed to be constant in the ground state, had to be adjusted downstream of the inhibition (Table S2, blue). Only the response of the alisertib treatment was predicted. For this purpose, based on the setting of the network for the HCC44 treated with ARS-1620, adjustments of assumed constant nodes were made downstream of the inhibition of the AURKA (Table S2, blue). Here we want to mention that the values in the simulation are activity values between 0 and 1 and are therefore not comparable to percentage values from the experimental measurements.

Specific adaptations of the *in silico* topology to simulate *in vitro* results: Importantly, in our new lung cancer model we considered both ERK1 and ERK2 as two separate signaling nodes to enable a more detailed regulation of EMT and proliferation connected to TGF-β. Furthermore, CD44 was integrated as an important co-regulator in central pathways.

[2] (B) Outcome (effector output): Experimentally measured parameters are shown in bold. All other nodes of the network were at zero activity at simulation start.
